# Low Hepatitis C Virus Prevalence among Men Who Have Sex with Men Attending Public Health Services in The Netherlands

**DOI:** 10.3390/v15122317

**Published:** 2023-11-25

**Authors:** Stephanie Popping, Sabine Haspels, Hannelore M. Gotz, W. C. J. P. M. van der Meijden, Mark van den Elshout, Bart J. Rijnders

**Affiliations:** 1 Department of Medical Microbiology and Infectious Diseases, Erasmus MC, University Medical Center, 3015 CN Rotterdam, The Netherlands; 2 Centre for Experimental and Molecular Medicine (CEMM), Amsterdam University Medical Centres—Location AMC, P.O. Box 22660, 1105 AZ Amsterdam, The Netherlands; 3 Department of Sexual Health, Public Health Service Rotterdam, 3011 EN Rotterdam, The Netherlands; 4 Department of Sexual Health, Public Health Service Gelderland-South, 4003 BW Tiel, The Netherlands; 5 Department of Sexual Health, Public Health Service of the Utrecht Region, 3521 AZ Utrecht, The Netherlands

**Keywords:** hepatitis C, MSM, hepatitis C elimination, micro-elimination

## Abstract

The hepatitis C virus (HCV) prevalence is high among men who have sex with men (MSM) with HIV in the Netherlands. Large reductions in HCV incidence among MSM with HIV, however, have occurred since treatment with direct-acting antivirals. Over the years, a broader understanding of the HCV epidemic has shown that HCV infections are not solely restricted to MSM with HIV, but they also occur among HIV-negative MSM. Currently, HCV testing among HIV-negative MSM is only provided for PrEP users and is not part of routine sexually transmitted infection (STI) screening among HIV-negative MSM who are not using PrEP. In this study, we screened 1885 HIV-negative MSM who did not participate in a PrEP program, with over 1966 STI screening visits at four different public health clinic sites. Among the 1885 MSM, only one person had a new HCV infection, resulting in a 0.05% (95% confidence interval 0.0–0.3) incidence. Based on our findings, we can conclude that systematic HCV testing at STI clinics may not yield significant benefits for this particular population.

## 1. Introduction

With the advent of highly effective direct-acting antivirals (DAAs), a sustained virological response has become within reach for virtually all hepatitis C virus (HCV)-infected individuals, thereby changing HCV from a chronic and potentially lethal infection into a curable disease [1,2]. Consequently, the World Health Organization (WHO) has set targets to eliminate HCV as a public health threat by the year 2030. These targets include a reduction in new HCV infections by 90% and a 65% reduction in HCV-related mortality. However, in 2020, an estimated 56·8 million people were infected with HCV worldwide [3].

In the Netherlands, the HCV epidemic was stable for years prior to widespread DAA use and was mainly concentrated among men who have sex with men (MSM) with HIV, with a 4.8% prevalence and 1.1% incidence [4,5]. These high numbers, however, were in stark contrast with the low overall HCV prevalence in the general Dutch population at 0.16–0.2%, one of the lowest worldwide [6,7]. In the Netherlands, MSM with HIV are generally accessing HIV care in one of the classified HIV treating centers. Therefore, this population is well defined and surveyed, allowing for micro-elimination (i.e., elimination targets obtained in a key population) of HCV as a first step in reaching the WHO elimination targets [8]. 

In order to achieve the WHO elimination goals, the Netherlands lifted all HCV treatment restrictions in 2015, resulting in the availability of DAAs for all newly diagnosed HCV infections. The rapid DAA uptake was followed by a substantial decrease in new HCV infections in the year thereafter among MSM with HIV. Furthermore, the incidence of HCV has continued to decline ever since, which was also seen in countries like the United Kingdom and Australia [9,10]. Nevertheless, HCV reinfections are common among MSM [4,11,12].

Over the years, a broader understanding of the HCV epidemic, however, has shown that having an HCV infection is not restricted to MSM with HIV. As such, HCV infections have, for instance, also been identified among HIV pre-exposure prophylaxis (PrEP) users and HCV transmission clusters, including MSM with and without HIV [13,14]. The risk of HCV has been associated with certain behaviors, such as unprotected receptive anal intercourse with multiple partners, the use of chemsex (using drugs for pleasure during sex), and fisting. Although these behavioral factors can also be present among HIV-negative MSM attending public health services (GGD: Gemeentelijke Gezondheidsdienst) for sexual transmitted infection (STI) screening, testing for HCV is currently not part of routine STI screening in these centers. Presently, routine HCV testing among HIV-negative MSM attending Dutch STI clinics is limited to those using PrEP or patients diagnosed with a lymphogranuloma venereum (LGV) infection. MSM with HIV instead are typically tested for HCV on a yearly basis at their HIV outpatient clinic. To reach the micro-elimination of HCV among MSM, regardless of their HIV status, we aimed to get a better estimate of the HCV prevalence among HIV-negative MSM who are not using PrEP and attending public health services for STI screening in the Netherlands. 

## 2. Methods

An HCV prevalence measurement was performed among 4 STI clinics (GGD Rotterdam, Utrecht, Arnhem, and Nijmegen) in 3 different regions (Rotterdam, Utrecht, and South Gelderland) from 2019 to the end of 2022. HIV-negative individuals who self-identified as MSM and did not use PrEP were informed about the possibility of being tested for HCV during a standard STI screening (including chlamydia, gonorrhea, syphilis, and HIV). STI screening through one of the GGD clinics is fully reimbursed for men who self-identify as MSM by the Dutch Ministry of Health, Welfare, and sport. HCV testing was performed at the GGD clinic using an HCV-IgG test (HCV-antibody) according to local laboratory protocols using one of the following systems: Liaison XL (DiaSorin, Saluggia, Italy), AxSYM HCV 3.0 (Abbott Laboratories, Abbott Park, IL, USA), or ARCHITECT Anti-HCV (Abbott Laboratories). HCV-positive IgG tests were subsequently validated through a secondary platform based on a more specific HCV IgG immunoblot. In cases where the HCV immunoblot confirmed a positive IgG test, an additional HCV-RNA test was performed using the Ap-tima HCV Quant Dx Assay (Hologic, San Diego, CA, USA). Individuals diagnosed with a new untreated HCV infection were referred for DAA treatment at a viral hepatitis center. 

From a convenience sample of 500 participants, results from a short questionnaire, including year of birth, reason for STI screening, and risk factors for HCV acquisition, were collected as well. All data were anonymized for our data analyzers at the STI clinics using study numbers. We reported median (interquartile ranges (IQR)) and 95% confidence intervals (CIs) calculated using the Wilson score interval. Data were analyzed using R version 4.1 (R Project for Statistical Computing). 

**Ethical statement:** The study was approved by the institutional review board of the Erasmus MC (protocol 2019-0105), and all participants provided informed consent

## 3. Results

In total, 1885 MSM were included and tested for HCV during 1966 STI clinic visits (2 visits *n* = 78, 3 visits *n* = 3). The median age was 31 (IQR 25–42) (Table 1). Around 43% of individuals had performed STI testing in the past. Of this group, 4% reported sex with a partner known to be HIV-positive. Risk factors for HCV acquisition were common; unprotected receptive intercourse was reported by 47% of MSM, 33% participated in group sex, 16% shared toys, and 5% had unprotected fisting in the past six months. None of the MSM reported slamming (injection drugs around sex), 15% of MSM shared straws or other objects for snorting drugs, and 5% tested positive for ulcerative STIs, such as syphilis, genital herpes, or LGV, in the past twelve months (Table 1). Among all the 1885 tested individuals, we detected three HCV antibody-positive MSM. At the time of HCV screening, no other STIs were detected. A further investigation showed that one of these three people had detectable HCV RNA. This MSM was therefore diagnosed with an active HCV infection. The remaining two MSM had no detectable HCV RNA and were diagnosed with a resolved HCV infection (Table 2). This resulted in an HCV 0.05% (95% confidence interval 0.0–0.3) incidence and an HCV prevalence of 0.05% (0.0–0.3) (Table 1). 

## 4. Discussion

Despite a high prevalence of behavioral risk factors previously found to be associated with HCV prevalence, we found a very low number of HCV infections and resolved HCV infections in HIV-uninfected MSM attending STI clinics in the Netherlands. As the main HCV epidemic in the Netherlands is assumed to be concentrated among HIV-infected MSM, and with the very high HCV treatment uptake in MSM with HIV, the very low HCV background prevalence is reassuring. Our low HCV prevalence rate is in line with the pooled HCV prevalence as described by Traeger et al. for 0.97% for HCV antibodies and 0.38% for HCV RNA prior to HIV PrEP use [15]. Together with the substantial decrease in HCV incidence among HIV-positive MSM in the Netherlands, these data show that HCV micro-elimination in the Dutch MSM population may be within reach. 

Currently, micro-elimination efforts within the Dutch MSM population focus on testing among high-risk subpopulations of MSM with HIV and those using PrEP. The results of our study support the continuation of these efforts, as our data suggest that broadening routine HCV screening to include routine STI screening among HIV-negative MSM not using PrEP would detect few new HCV infections (~0.05%). This is corroborated by a recent study conducted by Prinsenberg T et al., who describe an anonymous online validated home-based self-sampled testing service based on dried blood spot HCV-RNA assays. This service offers testing guidance to MSM who are at high risk using a validated metric to assess the risk of HCV [16,17]. A total of 44% of MSM received recommendations for HCV testing, and among this group of MSM, a high HCV-RNA positivity rate of 10.9% was found. This study highlights the presence of a subgroup exhibiting high-risk behavior, specifically seeking HCV testing. This emphasizes the importance of maintaining a targeted approach by focusing on subgroups within the MSM population exhibiting extremely high-risk behavior, rather than expanding HCV screening to those with lower-risk behavior attending STI clinics.

STI clinics in the Netherlands are free of charge, and care is decentralized and concentrated among the major cities. Although HCV-IgG screening is affordable, screening all MSM attending STI clinics might not be cost-effective with the current very low HCV background prevalence in the Netherlands. Reaching the ‘last mile’ of elimination is often very costly as there is a U-shaped cost curve (e.g., the end and the beginning of screening are expensive) [18]. However, untreated HCV can rapidly spread in the MSM community, resulting in increasing incidences. An alternative would be to focus on subgroups of people with continuous high-risk behavior who had a previous HCV infection, as reinfections remain high in the MSM population [12,17,19,20]. Additionally, the HCV epidemic among MSM in general should be tracked and extended to an international scale, as phylogenetic studies show a clear linkage between local HCV infections and European HCV clusters [21,22,23]. Phylogenetic analysis of outbreaks followed by contact tracing, targeted testing, and rapid treatment, preferably on a European level, could help further accelerate moving toward the ‘last mile’ and achieving the WHO elimination goals. 

Among the MSM attending the GGD for STI testing, we found a high number of behavioral risk factors, including condomless receptive anal intercourse, sex with multiple partners, and sharing of sex toys. Although the incidence of HCV infections was low, this does indicate the need for prevention strategies reducing other STIs, such as educational activities or behavioral counselling aiming at risk reduction. 

Our study has several limitations. First, due to the COVID-19 pandemic and the related restrictions on social life, the number of inclusions was smaller than the anticipated and desired number of inclusions. Moreover, we cannot exclude the fact that sexual risk behavior changed during the pandemic as well as the risk of an incident HCV infection. Second, the study was performed in four Dutch STI clinics outside the Amsterdam region, where sexual contacts may occur more frequently with partners from outside the Netherlands. In neighboring countries, the HCV prevalence may still be higher [24]. Lastly, as HCV testing was performed with an antibody-based test (HCV-IgG), we cannot exclude the fact that very recent HCV infections that are still in the seroconversion window remained undetected. 

In conclusion, our study observed a low prevalence of HCV among HIV-negative MSM who were not using PrEP across four STI clinics in the Netherlands. Therefore, our findings show that extending systematic HCV screening to STI clinics may not yield significant benefits for this particular population. Nevertheless, prevention strategies aimed at reducing risk behaviors are still in need for a population with several risk factors for STIs. 

## Figures and Tables

**Table 1:** Baseline characteristics and self-reported risk factors. Self-reported risk factors for HCV of MSM attending STI clinics among a convenience sample in the Netherlands (*n* = 500).

Clinical parameters	
Participants Median age in years (IQR)	*n* = 1885 31 (25–42)
**HCV status**	
Resolved HCV infection (95% CI) ^2^	2/1885 (0.1%, 0.0–0.4) ^1^
Active HCV infection (95% CI) ^3^	1/1885 (0.05%, 0.0–0.3) ^1^
Received a partner notification for STI testing	32%
Performed STI testing before	43%
**HCV risk factors over the past six months (available for *n* = 500)**	
Sex with an HIV-positive male—yes	4%
Sex with an HCV-positive male—yes	0%
Sex with multiple partners (group sex)	33%
Condomless receptive anal intercourse (bottom)	47%
Sharing of sex toys	16%
Unprotected fisting	5%
**HCV risk factors over the past twelve months (available for *n* = 500)**	
Injecting drug use (slamming)	0%
Sharing of straws or other objects when nasally administered drug	15%
Ulcerative sexually transmitted infection ^4^	5%

^1^ The 95% confidence interval is calculated using the Wilson score interval; ^2^ HCV antibody-positive with no detected HCV RNA; ^3^ HCV antibody-positive with detected HCV RNA. ^4^ Including syphilis, genital herpes, or lymphogranuloma venereum.

**Table 2:** Overview of patients with positive HCV antibody results during screening.

Patient	HCV IgG Antibody Test	HCV IgG Confirmation	HCV RNA	Conclusion
1	Positive	Positive	Positive	Active HCV infection
2	Positive	Positive	Negative	Resolved HCV infection
3	Positive	Positive	Negative	Resolved HCV infection

## Data Availability

The data presented in this study are available on request from the corresponding author. The data are not publicly available due to privacy reasons.

## References

[1] Zeuzem S., Foster G.R., Wang S., Asatryan A., Gane E., Feld J.J., Asselah T., Bourliere M., Ruane P.J., Wedemeyer H. (2018). Glecaprevir-Pibrentasvir for 8 or 12 Weeks in HCV Genotype 1 or 3 Infection. N. Engl. J. Med..

[2] Matthews G.V., Bhagani S., Van der Valk M., Rockstroh J., Feld J.J., Rauch A., Thurnheer C., Bruneau J., Kim A., Hellard M. (2021). Sofosbuvir/velpatasvir for 12 vs. 6 weeks for the treatment of recently acquired hepatitis C infection. J. Hepatol..

[3] Blach S., Terrault N.A., Tacke F., Gamkrelidze I., Craxi A., Tanaka J., Waked I., Dore G.J., Abbas Z., Abdallah A.R. (2022). Global change in hepatitis C virus prevalence and cascade of care between 2015 and 2020: A modelling study. Lancet Gastroenterol. Hepatol..

[4] Smit C., Boyd A., Rijnders B.J.A., van de Laar T.J.W., Leyten E.M., Bierman W.F., Brinkman K., Claassen M.A.A., den Hollander J., Boerekamps A. (2021). HCV micro-elimination in individuals with HIV in the Netherlands 4 years after universal access to direct-acting antivirals: A retrospective cohort study. Lancet HIV.

[5] Hullegie S.J., van den Berk G.E., Leyten E.M., Arends J.E., Lauw F.N., van der Meer J.T., Posthouwer D., van Eeden A., Koopmans P.P., Richter C. (2016). Acute hepatitis C in the Netherlands: Characteristics of the epidemic in 2014. Clin. Microbiol. Infect..

[6] Koopsen J., van Steenbergen J.E., Richardus J.H., Prins M., Op de Coul E.L.M., Croes E.A., Heil J., Zuure F.R., Veldhuijzen I.K. (2019). Chronic hepatitis B and C infections in the Netherlands: Estimated prevalence in risk groups and the general population. Epidemiol. Infect..

[7] Vriend H.J., Van Veen M.G., Prins M., Urbanus A.T., Boot H.J., Op De Coul E.L. (2013). Hepatitis C virus prevalence in The Netherlands: Migrants account for most infections. Epidemiol. Infect..

[8] Lazarus J.V., Safreed-Harmon K., Thursz M.R., Dillon J.F., El-Sayed M.H., Elsharkawy A.M., Hatzakis A., Jadoul M., Prestileo T., Razavi H. (2018). The Micro-Elimination Approach to Eliminating Hepatitis C: Strategic and Operational Considerations. Semin. Liver Dis..

[9] Doyle J.S., van Santen D.K., Iser D., Sasadeusz J., O’Reilly M., Harney B., Traeger M.W., Roney J., Cutts J.C., Bowring A.L. (2021). Microelimination of Hepatitis C Among People With Human Immunodeficiency Virus Coinfection: Declining Incidence and Prevalence Accompanying a Multicenter Treatment Scale-up Trial. Clin. Infect. Dis..

[10] Garvey L.J., Cooke G.S., Smith C., Stingone C., Ghosh I., Dakshina S., Jain L., Waters L.J., Mahungu T., Ferro F. (2021). Decline in Hepatitis C Virus (HCV) Incidence in Men Who Have Sex With Men Living With Human Immunodeficiency Virus: Progress to HCV Microelimination in the United Kingdom?. Clin. Infect. Dis..

[11] Boerekamps A., van den Berk G.E., Lauw F.N., Leyten E.M., van Kasteren M.E., van Eeden A., Posthouwer D., Claassen M.A., Dofferhoff A.S., Verhagen D.W.M. (2018). Declining Hepatitis C Virus (HCV) Incidence in Dutch Human Immunodeficiency Virus-Positive Men Who Have Sex With Men After Unrestricted Access to HCV Therapy. Clin. Infect. Dis..

[12] Martinello M., Carson J.M., Van Der Valk M., Rockstroh J.K., Ingiliz P., Hellard M., Nelson M., Lutz T., Bhagani S., Kim A.Y. (2023). Reinfection incidence and risk among people treated for recent hepatitis C virus infection. AIDS.

[13] Hoornenborg E., Achterbergh R.C.A., Schim van der Loeff M.F., Davidovich U., Hogewoning A., de Vries H.J.C., Schinkel J., Prins M., van de Laar T.J.W., Amsterdam PrEP Project Team in the HIV Transmission Elimination Amsterdam Initiative (2017). MSM starting preexposure prophylaxis are at risk of hepatitis C virus infection. AIDS.

[14] Popping S., Cuypers L., Claassen M.A.A., van den Berk G.E., De Weggheleire A., Arends J.E., Boerekamps A., Molenkamp R., Koopmans M.P.G., Verbon A. (2022). Persistent Transmission of HCV among Men Who Have Sex with Men despite Widespread Screening and Treatment with Direct-Acting Antivirals. Viruses.

[15] Traeger M.W., Harney B.L., Sacks-Davis R., van Santen D.K., Cornelisse V.J., Wright E.J., Hellard M.E., Doyle J.S., Stoové M.A. (2023). Incidence and Prevalence of Hepatitis C Virus Among HIV-Negative Gay and Bisexual Men Using HIV Pre-exposure Prophylaxis (PrEP): A Systematic Review and Meta-analysis. Open Forum Infect. Dis..

[16] Newsum A.M., Stolte I.G., van der Meer J.T., Schinkel J., van der Valk M., Vanhommerig J.W., Buve A., Danta M., Hogewoning A., Prins M. (2017). Development and validation of the HCV-MOSAIC risk score to assist testing for acute hepatitis C virus (HCV) infection in HIV-infected men who have sex with men (MSM). Eurosurveillance.

[17] Prinsenberg T., Schinkel J., Zantkuijl P., Davidovich U., Prins M., van der Valk M. (2022). Internet-guided HCV-RNA testing: A promising tool to achieve hepatitis C micro-elimination among men who have sex with men. J. Viral Hepat..

[18] Nichols B.E., Girdwood S.J., Crompton T., Stewart-Isherwood L., Berrie L., Chimhamhiwa D., Moyo C., Kuehnle J., Stevens W., Rosen S. (2019). Monitoring viral load for the last mile: What will it cost?. J. Int. AIDS Soc..

[19] Popping S., Nichols B., Rijnders B., van Kampen J., Verbon A., Boucher C., van de Vijver D. (2019). Targeted HCV core antigen monitoring among HIV-positive men who have sex with men is cost-saving. J. Virus Erad..

[20] Garvey L., Cooke G.S. (2022). Engaging with HCV reinfection to advance microelimination. Lancet HIV.

[21] Koopsen J., Parker E., Han A.X., van de Laar T., Russell C., Hoornenborg E., Prins M., van der Valk M., Schinkel J. (2021). Hepatitis C Virus Transmission Among Men Who Have Sex With Men in Amsterdam: External Introductions May Complicate Microelimination Efforts. Clin. Infect. Dis..

[22] Popping S., Verwijs R., Cuypers L., Claassen M.A., van den Berk G.E., De Weggheleire A., Arends J.E., Boerekamps A., Molenkamp R., Koopmans M.P. (2020). Transmission of NS5A-Inhibitor Resistance-Associated Substitutions Among Men Who Have Sex With Men Recently Infected with Hepatitis C Virus Genotype 1a. Clin. Infect. Dis..

[23] Koopsen J., Matthews G., Rockstroh J., Applegate T.L., Bhagani S., Rauch A., Grebely J., Sacks-Davis R., Ingiliz P., Boesecke C. (2023). Hepatitis C virus transmission between eight high-income countries among men who have sex with men: A whole-genome analysis. Lancet Microbe.

[24] Cortesi P.A., Fornari C., Conti S., Antonazzo I.C., Ferrara P., Ahmed A., Andrei C.L., Andrei T., Artamonov A.A., Banach M. (2023). Hepatitis B and C in Europe: An update from the Global Burden of Disease Study 2019. Lancet Public Health.

